# Facial Rejuvenation with Concentrated Lipograft—A 12 Month Follow-Up Study

**DOI:** 10.3390/cells10030594

**Published:** 2021-03-08

**Authors:** Lukas Prantl, Eva Brix, Sally Kempa, Oliver Felthaus, Andreas Eigenberger, Vanessa Brébant, Alexandra Anker, Catharina Strauss

**Affiliations:** Department of Plastic, Aesthetic and Reconstructive Surgery, University of Regensburg, Franz-Josef-Strauss-Allee 11, D-93053 Regensburg, Germany; lukas.prantl@ukr.de (L.P.); eva.brix@ukr.de (E.B.); sally.kempa@ukr.de (S.K.); oliver.felthaus@ukr.de (O.F.); andreas.eigenberger@ukr.de (A.E.); vanessa.brebant@ukr.de (V.B.); Alexandra.anker@ukr.de (A.A.)

**Keywords:** adipose-derived stem cells, facial rejuvenation, patient’s satisfaction, fat grafting, stromal vascular fraction

## Abstract

Lipofilling is a popular technique to treat volume loss in aging patients. The isolated adipose tissue is composed of adipocytes and stromal vascular fraction cells, which include adipose-derived stem cells (ASC). We hypothesize that the patient’s wrinkle severity scale (WSS) and patient’s satisfaction on the global aesthetic improvement scale (GAIS) can be improved after using concentrated lipoaspirate. Fourteen patients (54 years ± 11.09 years) with volume loss in the midface area underwent waterjet-assisted liposuction (Human Med AG, Schwerin, Germany). Fat was centrifuged in an ACP Double Syringe (Arthrex GmbH, Munich, Germany) using Rotofix 32A centrifuge (Andreas Hettich, GmbH & Co.KG, Tuttlingen, Germany). Homogenization was performed using the double syringe and a 1.4 mm female–female luerlock connector. After a second centrifugation, patients received periorbital (PO) and nasolabial (NL) lipografting. ASC count was performed after enzymatical digestion. Vitality of cells was assessed using a resazurin assay. During long-term follow up (12 months, *n* = 10), we found a high patient’s satisfaction (GAIS 1+/−0.52) and a good improvement of the WSS during short- and long-term follow-up. The ASC count of processed lipoaspirate was 2.1-fold higher than of unprocessed lipoaspirate (*p* < 0.001). The difference of ASC in sedimented and simply centrifuged lipoaspirate was also significant (*p* < 0.05). Facial rejuvenation with concentrated fat graft offers good results concerning objective aesthetic outcome and patient’s satisfaction.

## 1. Introduction

Lipofilling, the autologous transplantation of adipose tissue, has been used since the end of the 19th century, especially for posttraumatic or congenital defects. Currently, lipofilling has become a popular technique to treat volume and contour abnormalities in aesthetic and reconstructive surgery. The processed fatty tissue can be seen as a natural filler instead of commercially available products [1,2]. Fat grafting is used widely in clinical practice for various indications, but there are many variations on fat harvesting, preparation, and reinjection [3,4,5,6]. Facial plastic surgery, for reconstructive and cosmetic reasons, aims to achieve harmony of facial features in case of volume loss, aging or just as an aesthetic tool. Lipofilling promises to establish a custom fit long-term outcome with minor adverse effects as opposed to the more severe adverse effects that temporary fillers such as hyaluronic acid filler may cause [7,8]. Autologous fat grafts offer several benefits, including lack of immunogenicity, simplicity of surgical procedure, low cost, and easy accessibility. Furthermore, it has become increasingly common to utilize fat that has been cryopreserved after the initial fresh fat graft for potential future application [9]. Fat grafts are harvested from a region that is generally more abundant and injected into a secondary site.

The initial isolated adipose tissue is composed of adipocytes and stromal vascular fraction cells, which include adipose-derived stem cells (ASCs), preadipocytes, fibroblasts, vascular endothelial cells, and a variety of immune cells [10]. ASCs, which are cells of mesenchymal origins, are believed to be responsible for not only improving tissue contour of dermal and subdermal tissue but also weakening scar adhesions and paling the scars themselves. Preclinical studies suggested that ASC live longer than normal fat cells. ASCs offer a great regenerative potential for angioneogenesis, cell differentiation, and cell proliferation [11]. The beneficial effects to fat graft survival are believed to depend on secreted factors [12,13]. A higher stem cell percentage resulting in a higher growth factor secretion at the grafting site might be able to overcome the problem of volume losses of up to 70 percent [14,15]. The development of fine harvesting and injection cannulas made it possible to inject small adipose tissue particles in small volume areas, such as the face [16]. Liposuction should be performed gently with special cannulas under continuous negative pressure to guarantee maximal vitality and purity of adipocytes [17,18,19]. To achieve maximum viability in lipotransfer the optimal vacuum pressure for liposuction is between −0.5 and −0.55 bar [20,21].

To properly address volume loss rates which vary up to 70% [14] volume deficits are often overcorrected. This may result in further surgeries and patient’s dissatisfaction with the results. The main surgical goal seems to be increasing of uptake rate while keeping adverse effects at a low rate and to achieve a stable long-term result immediately after the treatment.

Several studies have proven processing of lipoaspirate can increase healing rate [22,23], but there is still no universal agreement of how one should harvest, process, or graft fat [24].

Accurate photographic documentation has become essential in reconstructive and aesthetic plastic surgery both for clinical and scientific purposes. Obtaining standardized, consistent, and relevant digital images is not easy outside a photographic studio [25,26]. Furthermore, the assessment of the outcome often seems subjective while digital photography remains a main tool for visualization of results [27].

Additionally, for objective evaluation of treatment outcome several tools were developed as 3D photographs, which show the volume uptake in several parts of the face or the reduction of wrinkles. Satisfaction of patients with aesthetic procedures can easily be monitored by using the global aesthetic improvement scale (GAIS) [28]. The wrinkle severity scale (WSS) is a helpful tool for physicians to monitor the success of antiaging interventions [29]. It is quite popular for facial fat grafting not only among plastic surgeons but also dermatologists and craniofacial surgeons.

We hypothesize that using centrifugated and concentrated lipoaspirate improves patient’s wrinkle severity scale (WSS) and patient’s satisfaction (GAIS).

## 2. Materials and Methods

### 2.1. Patient Demographics Informed Consent

A total of twelve healthy, tumor-free women and two tumor-free man aged on average 54 years ± 11.09 years (*n* = 14; range from 30 to 72 years) underwent first water-assisted liposuction at different regions of the body (abdominal, flanks, hips or legs) and after lipoaspirate processing, fat grafting for volume loss in nasolabial fold (NLF) and periorbital (PO) (Table 1).

Interventions have been approved by the Ethics Committee of the University Hospital of Regensburg (No.17-520-101). All patients agreed to explained surgical intervention (liposuction, lipoaspirate processing and facial lipofilling for nasolabial or periorbital folds) and documentation by digital and 3D photography by signature. Inclusion criteria were any kind of volume loss in midface area.

Exclusion criteria were additive filler therapy or midface surgery performed. 4 patients missed follow up and were excluded in long-term follow-up analysis.

### 2.2. Liposuction Technique

All patients received a waterjet-assisted liposuction. In preparation for harvesting lipoaspirate, a 0.9% (weight/volume) solution of sodium chloride containing adrenaline at a concentration of 1:200.000 was infiltrated over approximately 15 min by means of a 2.5-mm injection cannula (Human Med AG, Schwerin, Germany). The volume was equal to the volume of fat tissue harvested. The lipoaspirate was harvested using 3.8-mm cannulas (Human Med AG, Schwerin, Germany) and the Body-Jet (Human Med AG), which allows a water jet–assisted liposuction. This medicinal product ensures an even negative pressure of less than 0.5 mbar.

### 2.3. Processing of Lipoaspirate

Lipoaspirate is extracted from solution in suction container (Human Med AG) after sedimentation and filling into 15 mL double syringe (Arthrex ACP Double Syringe System, Munich, Germany). Centrifugation is performed at 2500 rpm for 4 min (Rotofix 32 A, Andreas Hettich GmbH & Co.KG, Tuttlingen, Germany) to separate fat, oil, and tumescent solution, a three-layer can be seen in double syringe afterward.

**Figure 1:**
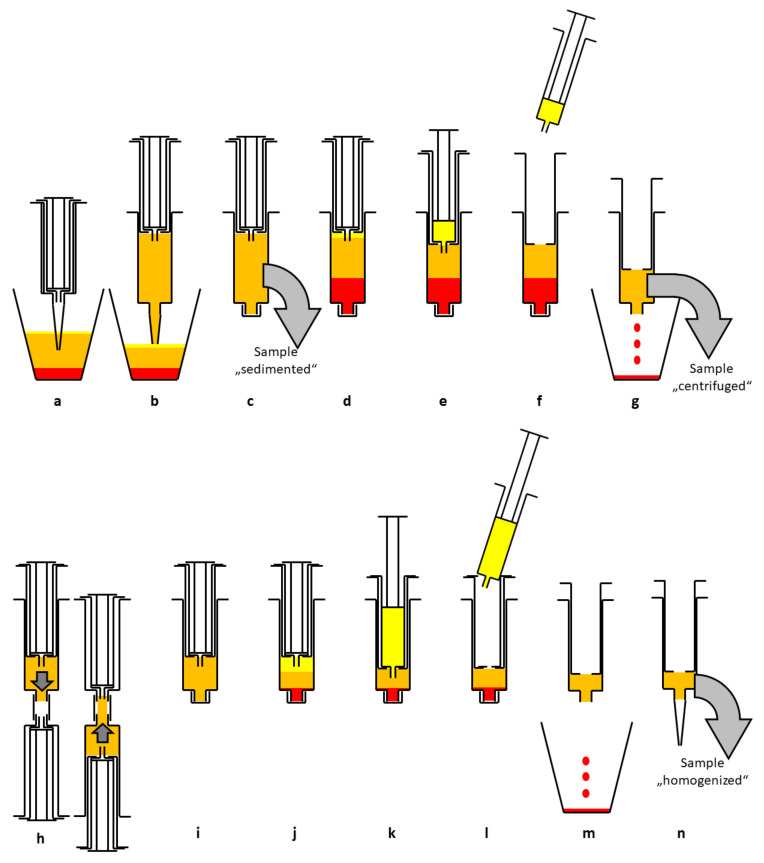
Schematic diagram of the enrichment process. After sedimentation in the suction container (**a**) lipoaspirate is transferred to a 15 mL double syringe (**b**). The sample “sedimented” is taken for analysis (**c**). After centrifugation (2500 rpm, 4 min) three layers can be seen (**d**). The upper oil phase is transferred to the small inner syringe (**e**) and discarded (**f**). The blood and tumescent solution are discarded as well (**g**). The sample “centrifuged” is taken for analysis. The syringe is connected to a Tulip-1.4-mm connector and another syringe and the lipoaspirate is emulsified by forcing it through the connector at least 10 times (**h**). The now homogenized lipoaspirate (**i**) is centrifuged again at 2500 rpm for 4 min, resulting in three layers (**j**). The upper oil phase from disrupted adipocytes is transferred to the small inner syringe (**k**) and discarded (**l**). The aqueous phase is discarded, too (**m**). The remaining lipoaspirate contains a high percentage of ASCs and is ready for lipofilling (**n**). The sample “homogenized” is taken for analysis.

After extracting the oil into smaller inner syringe, the aqueous phase at the bottom of the syringe is discarded. The following emulsification and homogenization step is done by using a Tulip-1.4-mm connector (Tulip Medical Products, San Diego, CA, USA) attached to a second syringe. The lipoaspirate was forced manually in both directions with high velocity through the double syringes. After a second centrifugation at 2500 rpm for 4 min, the released lipids were removed by transfer in the inner syringe by slowly pushing down on the outer syringe while slowly pulling up the plunger of the small inner syringe (see Figure 1)

**Figure 2:**
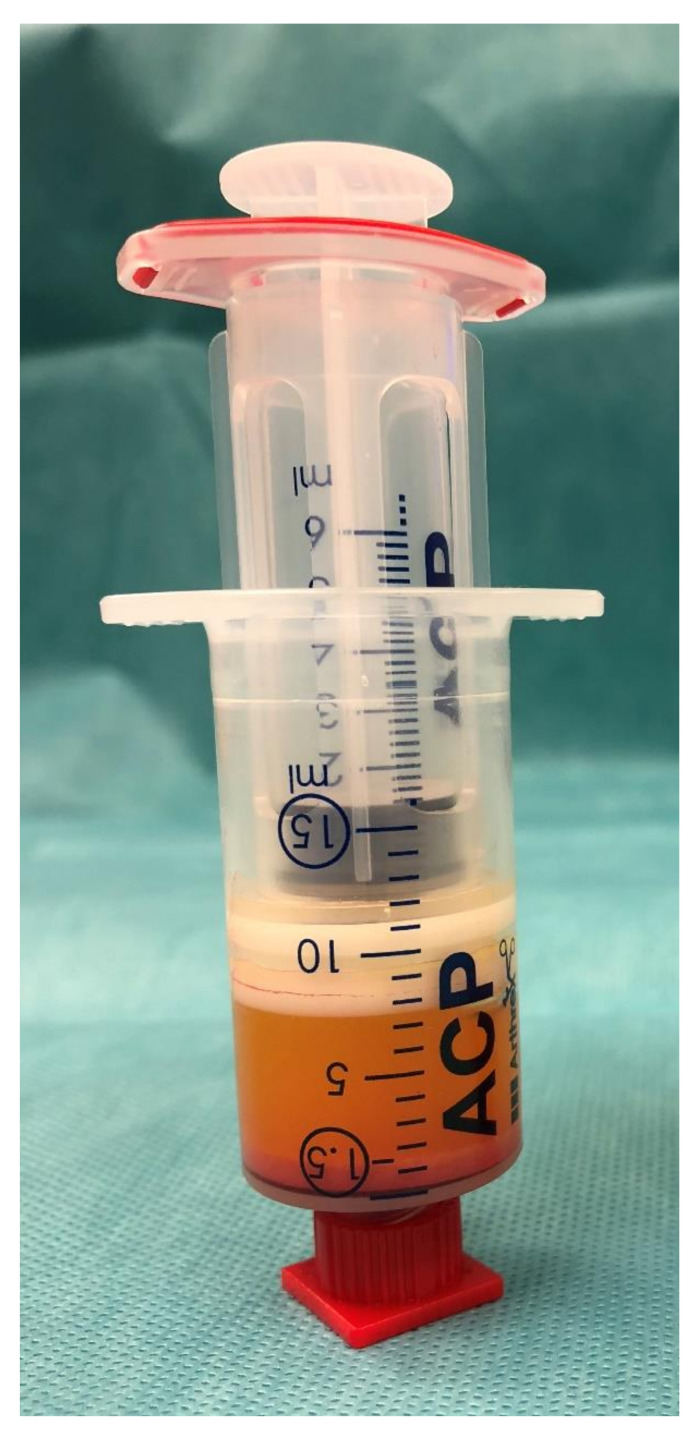
Emulsified, homogenized fat graft with oily phase and cell pellet at the bottom.

This emulsified, homogenized fat graft (about 1.5 mL) was transferred to 1 mL syringes via the 2.4- or 1.4-mm transfer device (see Figure 2).

### 2.4. Stem Cell Isolation and Counting

Sedimented, centrifuged, and emulsified, homogenized lipoaspirate was enzymatically digested as described previously [10]. Briefly, 1 mL of α-MEM (Gibco (Thermo Scientific, Waltham, MA, USA) containing 0.2% (*w*/*v*) collagenase (from Clostridium histolyticum, Sigma Aldrich, St. Louis, MO, USA) was added to 1 mL of lipoaspirate. Samples were incubated for 45 min at 37 °C under constant agitation and centrifuged at 500 rpm for 5 min, subsequently. The supernatant was discarded, and the cells were resuspended in cell culture medium. After a small volume was set aside for the vitality assay, the cells were seeded into T25 cell culture flasks and grown for 3 days at 37 °C and 5% CO2 in a humidified atmosphere. Plates were washed every day to remove nonadherent cells. The plastic-adherent cells were counted at five randomly chosen sites (1 mm^2^ each). The total cell number was calculated using the total cell culture area (25 cm^2^).

### 2.5. Cell Vitality Assay

Vitality of cells isolated from sedimented, centrifuged, and homogenized lipoaspirate was evaluated using a resazurin assay. The cells set aside after isolation were seeded in 96-well plates in quintuplicates. For measurement, cell culture medium was supplemented with 0.07 µM resazurin (Sigma Aldrich). Metabolic conversion of resazurin into the fluorescent resorufin was detected using a multiwell plate reader (VarioScan, Thermo Scientific).

### 2.6. Flow Cytometry

Three days after seeding cells were washed with PBS (phosphate-buffered saline, PAALaboratories, Pasching, Austria) and detached by incubation with 500 µL Trypsin/EDTA (Promo-Cell) for 5 min at 37 °C. Cells were distributed to FACS tubes. After centrifuging (300 rpm, 5 min) the supernatant was removed and the cells were resuspended in 40 µL staining buffer consisting of PBS containing 0.01% sodium azide, 0.5% BSA, and 2 nM EDTA. Then 5 µL of CD44 antibody or isotype control antibody (Alexa-Fluor488 antimouse/human CD44 Clone IM7 or Alexa-Fluor488 mouse IgG1 isotype control (FC) Clone MOPC-21. BioLegend) were added and the cells were incubated on ice in the dark for 1 h. After addition of 1 mL staining buffer, the cells were centrifuged and the supernatant was removed. Cells were resuspended in 500 µL staining buffer and measured using the FACS Canto II (BD Biosciences, Heidelberg, Germany). At least 50,000 events of each sample were recorded.

### 2.7. Lipofilling Technique

Fat grafts which resulted after homogenization and two rounds of centrifugation (see 2.3) (1–3 mL) were injected into nasolabial folds and/or periorbital folds (within subdermal layer similar a filler, subcutaneous tissue and under superficial musculoaponeurotic system) in multiple tissue planes, tunnels, areas with a syringe connecting with a blunt needle whose external diameter is 1 or 1.5 mm and 2 mm. For additional volume effect homogenized fat (after one centrifugation and homogenization with intersyringe shifting) was also used. For the subdermal/dermal injection, a 20 G needle (0.603 mm) was used. Care was taken to inject fat grafts in only small quantities in one place each time (maximum: 3 mL), radially from distal to proximal. The syringe was drawn back before each injection to check blood return to avoid hematoma or to inject fat grafts into blood vessels.

In the periorbital area, extra care was taken to prevent intra-arterial injection. Gentle massage was done with finger or palm of the surgeon to ensure a smooth correction.

### 2.8. Digital Photography

Documentation of patient’s face was done preoperatively and at 3 months, 6 months and 12 months after lipofilling using a digital photograph Nikon Lumix NR/DMC-5272 (Nikon Corporation, Tokyo, Japan). Setting takes place while patient standing in upright position. Illumination was done by two lamps at 45° with respect to the patient on a plane parallel to frontal lamp. A minimum of five pictures needed to be taken: frontal view, oblique view (left/right) and lateral view (right/left).

### 2.9. 3D Photography and Wrinkle Severity Rating Scale

Documentation of the patient’s face was done preoperatively and at 3 months, 6 months and 12 months after lipofilling using a 3D skin camera (Antera 3D^®^, Miravex Limited, Ireland) which enables the user to identify skin surface, wrinkles or hyperpigmentations. The camera is used for each region (nasolabial fold or periorbital) in three different angles: frontal view; oblique vies (45 degrees) and lateral view.

**Figure 3:**
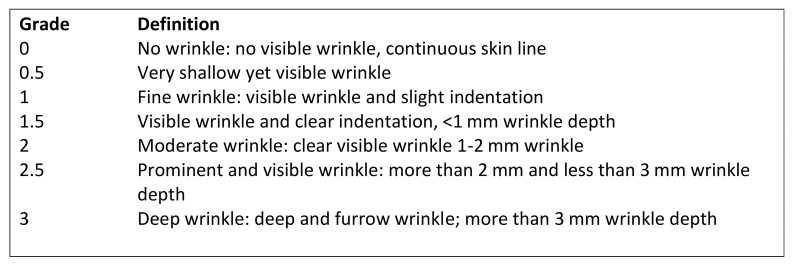
Wrinkle severity scale for nasolabial or periorbital folds (WSS).

The depth of the facial folds was measured using the modified wrinkle severity scale by Fitzpatrick [24,25] which has a wide use in clinical examination (Figure 3). Pre- and postoperative fold measurements were contrasted and compared.

### 2.10. Evaluation of Patient’s Satisfaction

**Figure 4:**
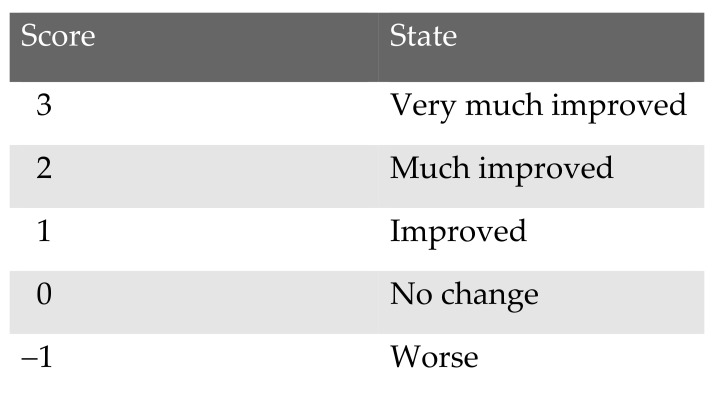
Patient’s satisfaction measured by global aesthetic improvement scale (GAIS).

To evaluate patient’s subjective appreciation of the surgical procedure the global aesthetic improvement scale (GAIS) was used (Figure 4) The documentation was primary 6 weeks after surgical intervention and at least at 9 to 12 months after surgical intervention (long term follow up).

### 2.11. Statistical Analysis

The unpaired students *t*-test was used to compare the pre- and postoperative measurements and the patient’s satisfaction. Values of *p* < 0.05 were considered statistically significant (*: *p* value < 0.05; **: *p* value < 0.01; ***: *p* value < 0.001).

## 3. Results

### 3.1. Cell Isolation

**Figure 5:**
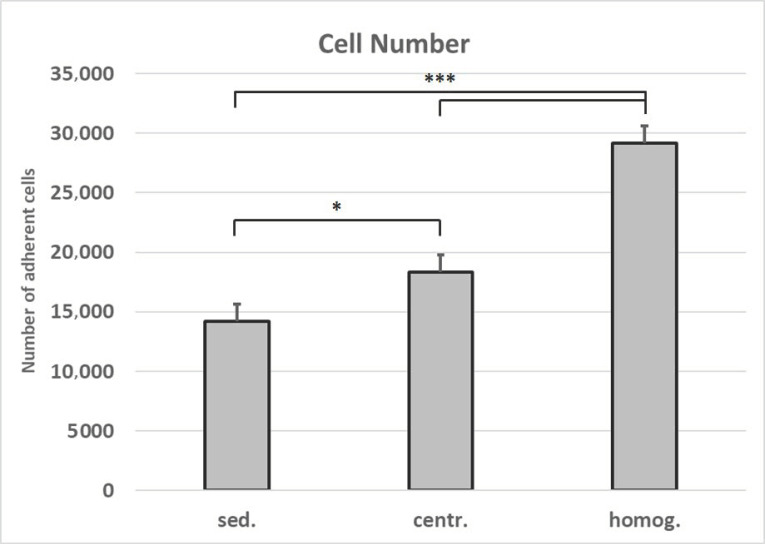
Number of cells isolated from varying tissue processings. Mean values and standard deviations are shown. Student’s test was used to assess statistical significance (* *p* < 0.05; *** *p* < 0.001). (sed.: sedimented; centr.: centrifuged; homog.: homogenized).

**Figure 6:**
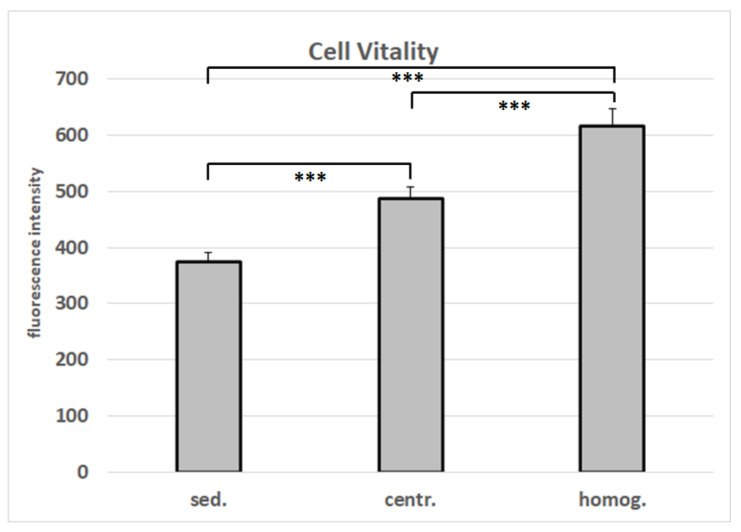
Vitality of cells isolated from varying tissue processings. Mean values and standard deviations are shown. Student’s *t*-test was used to assess statistical significance (*** *p* < 0.001). (sed.: sedimented; centr.: centrifuged; homog.: homogenized).

The number of adherent cells isolated after homogenization and centrifugation from 1 mL lipoaspirate is 2.1-fold higher than the number of cells that were isolated from 1 mL of unprocessed tissue (Figure 5). This increase in cell yield is verified by the data from the cell vitality assay (Figure 6).

**Figure 7:**
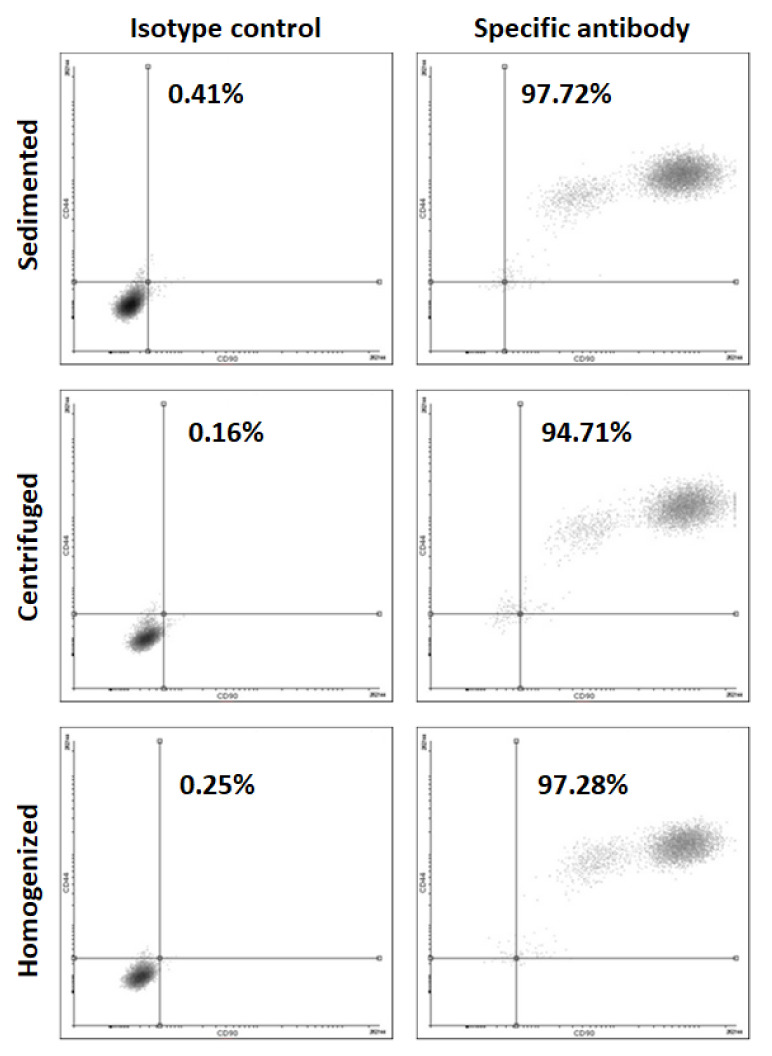
Flow cytometry for MSC marker CD 44 for the cells isolated from varying tissue processings.

The cells isolated from the differently processed tissues are positive for MSC marker CD44 (Figure 7).

### 3.2. Lipofilling Volume

In 13 patients concentrated graft was used for NLF with mean volume (cc) of 2.5 cc ± 1. 38 cc (*n* = 13; range 1 to 5 cc). Lipofilling in periorbital (PO) region was done in 14 patients, the mean volume was 2.21 cc ± 1.01 cc (*n* = 14; range 1–5 cc) (Table 1).

### 3.3. Improvement of Wrinkle Severity Scale (WSS)

**Figure 8:**
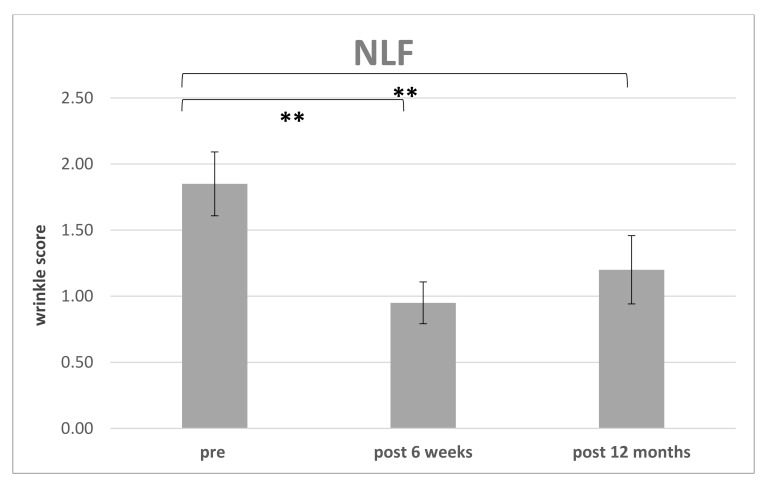
Improvement of the wrinkle severity scale related to the nasolabial fold (NLF): Comparison of WSS preoperative to 6 weeks and 12 months postoperative (** *p* < 0.01).

**Figure 9:**
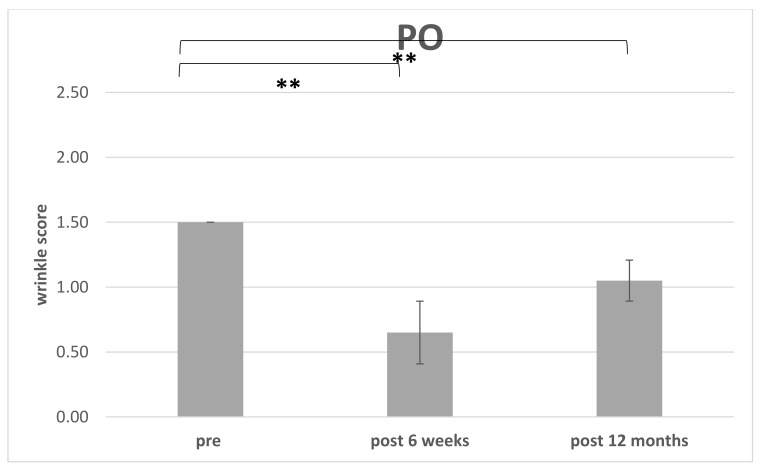
Improvement of the wrinkle severity scale related to the periorbital fold (PO): Comparison of WSS preoperative to 6 weeks and 12 months postoperative (** *p* < 0.01).

The improvement of WSS preoperative for each region (PO or NLF) were compared to postoperative at 6 weeks (*n* = 14) (Table 2) and the endpoint was defined after 12 months (*n* = 10) (Table 3) (Figure 8 and Figure 9). 4 patients missed the endpoint because of additional surgical interventions and/or missing follow up.

The preoperative WSS for NLF was 1.81 ± 0.24 (*n* = 14; range: 1.5–2) and after 6 weeks an improvement was seen with following scores: WSS for NLF 0.92 ± 0.18 (*n* = 14; range: 0.5–1).

### 3.4. Patient’s Satisfaction with Surgical Intervention (Global Aesthetic Improvement Scale)

The satisfaction with the surgical interventions was measured by global aesthetic improvement scale (GAIS). The outcome did not differ after 6 weeks in comparison to 12 months follow up and was 1 ± 0.52 (*n* = 10; range 0–2) (Table 2 and Table 3).

**Figure 10:**
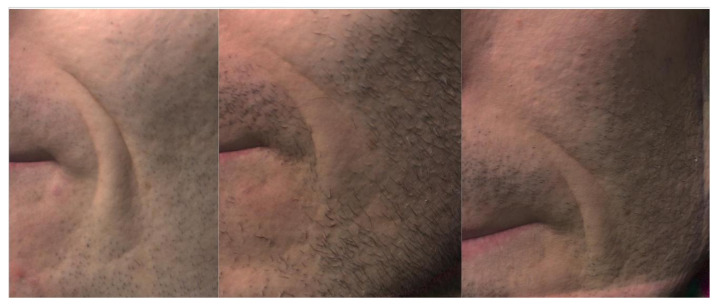
Comparison of preoperative and postoperative photography based on the nasolabial fold (patient no. 6); **Left**: NLF preoperative (WW 2); **middle**: NLF after injection of 1,5 cc after 6 weeks (WWS 1); **right**: NLF after 12 months (WWS 1.5)

Patient no. 6 (Table 3) suffered in particular from deep nasolabial fold (WSS 2) with overall slim appearance. Six weeks after injection of 1.5 cc per each side a WSS of 1 was reached. After total reduction of swelling, a final WSS of 1.5 was reached. The facial appearance was much improved (GAIS = 2). The photo documentation as well as the 3D photography (Figure 10) show an improvement of the wrinkle depth over time.

## 4. Discussion

Our study demonstrates that autologous fat grafting with mechanical processed lipoaspirate helps to achieve good long-term results in patients with volume deficits and wrinkles in midface and periorbital areas concerning both patient’s and surgeon’s evaluation. It is an easy, safe and quick procedure with highly satisfied patients in a rather demanding patient cohort. During long-term follow up patient’s satisfaction (after 12 months) reached a mean of 1.0+/−0.47 (mean+/−standard deviation) on the global aesthetic improvement scale (GAIS). Short-term follow-up showed a slightly higher patient’s satisfaction of 1.07+/−0.47 (mean+/−standard deviation SD) on the GAIS (compare Table 2 and Table 3).

Initially, there was a drop in the wrinkle severity rating scale from 1.8 to 0.95 (mean) for the nasolabial fold and respectively from 1.5 to 0.65 for the periorbital region after 6 weeks postoperative. We then observed an increase in the WSS to 1.2 (mean) in the nasolabial fold and to 1.05 in the periorbital region during long-term follow-up (see Table 3). We assume that the initial drop was caused by the intervention itself. It might be intensified by postoperative swelling and edema which usually vanishes after 2–4 weeks (see Figure 9). The increase during long-term follow-up might be due to natural aging processes, but sun exposition or weight fluctuation could also cause synergistic effects. Sun exposition is difficult to track but weight should be recorded during follow-up.

There are many clinical studies which focus on surgeon’s evaluation of effects but there are only a few which also focus on patient’s satisfaction. In 2013, Mailey et al. compared patient’s satisfaction with autologous fat transfer or cell-enriched fat transfer (mean follow-up 10.7 months) and found only a difference between both groups concerning satisfaction with skin pigmentation [30]. However, in this study, the Celution^®^ system (Cytori Therapeutics, San Diego, USA) was applied and the stromal vascular fraction was isolated using a proteolytic collagenase enzyme reagent (Celase^®^; Cytori Therapeutics). The cells were then washed to remove residual enzyme and concentrated within the closed automated system in the operating room. Total time for the digestion and enrichment process was approximately 1.5 h. The authors also reported a high dropout rate as only 17 of 36 patients completed the mailed satisfaction survey. Wang et al. used autologous fat graft to restore midface fat compartments and achieved patient’s satisfaction in 95.2% [31]. They evaluated patient’s satisfaction on a tripartite rating scale (satisfied—mostly satisfied—not satisfied).

Among our patients three received additional surgical interventions such as lower blepharoplasty or a midface lift (*n* = 1 lower blepharoplasty, *n* = 2 facelift). Periorbital fat grafting was especially useful in combination with midface lift as this region would otherwise not be addressed properly. These patients were highly satisfied with the procedures (GAIS 2) and achieved good results on the WSS (periorbital 1.5, nasolabial 1). Others also recommend augmenting surgical interventions such as lower blepharoplasty or midface lift with enriched fat grafting [32,33]. Marten et Ellyassnia even believe fat grafting to be the “missing link” in facial rejuvenation to address the loss of volume which cannot be treated by facelift alone [34].

Generally, the patient’s satisfaction should be elevated with all aesthetic procedures to get to know the demands and needs of this frequently challenging patient population even better.

### 4.1. Mechanical Processing of Lipoaspirate Versus Enzymatic Processing

Especially in Europe, there has been an intense discussion about the safety aspects of fat grafting due to enzymatic processing [35,36]. Our results show that mechanical enriched adipose transfer also offers reliable and sufficient results without risking a breach of legal restrictions. The use of enzymes (collagenase) and the risk of enzyme residues in the fat graft can be avoided.

There have also been apprehensions that mechanical processing might affect the genome and therefore protein synthesis in adipose cells. An analysis of cells in lipoaspirate after mechanical processing showed that mechanical processing does not affect the secretome of autologous adipose cells and can therefore be used safely in a multivariety of surgical settings [37]. On top, the authors managed to show that mechanical processing was able to boost the cellular fraction in comparison to nonprocessed lipoaspirate. This confirmed the results of Ibatici et al. [38].

In this study, we were able to demonstrate that the number of adherent cells isolated after homogenization and centrifugation from 1 mL lipoaspirate is 2.1-fold higher than the number of cells that were isolated from 1 mL of unprocessed tissue (Figure 2). This pellet contains significantly higher ASCs or respectively mesenchymal stem cells (MSC) than nonprocessed lipoaspirate [39].

It is quite challenging to compare the results of clinical studies using fat grafting as there are not only multiple techniques (conventional versus enzymatically/mechanically enriched substrate) but also quite some confusing terminology.

Many authors use the term stromal vascular fraction (SVF) to describe a cell enriched layer/pellet after decantation, centrifugation and intersyringe shifting [32]. This pellet contains significantly higher numbers of ASCs or mesenchymal stem cells (MSC), respectively than nonprocessed lipoaspirate [39]. To name it SVF, there theoretically needs to be an enzymatic digestion process which leads to the additional term “mechanical stromal vascular fraction enriched lipotransfer” [40]. These only vaguely correct but liberally used terms and definitions complicate interpretation and comparison of clinical trials even more [41,42].

We demonstrate that lipofilling with mechanical processed autologous fat graft shows positive long-term effects due to the increased ASC concentration. Our results are in line with those of other studies [11,40,43]. Tonnard et al. emulsified and filtered lipoaspirate to diminish adipocytes and fiber concentration and to enrich stem cells and growth factors and established the term “nanofat” for this transplant material [44]. However, we used a slightly different approach without filtering. Yin et al. performed a clinical study with 50 patients comparing results of fat grafting with versus without enriched liposubstrate. The survival rate of SVF-enriched fat grafts was significantly higher than that of control grafts [43]. Another clinical study also focused on decantation, washing and centrifugation of lipoaspirate to increase the cellular fraction and achieved satisfying aesthetic results [40]. We were able to obtain far more ASCs than the 1600 ASCs counted in 1 mL fat on average (see Figure 4) in their study, but these results are difficult to compare due to much diverging processing protocols. We incubated the processed fat for three days and focused on CD-44 positive mesenchymal stem cells (see Figure 6).

### 4.2. Limitations

There was a high dropout rate in our patient cohort during long-term follow up (>6 weeks). In *n* = 4 this was related to missing follow-up appointments. A high dropout rate is a common problem of many clinical studies, Mailey et al. authors also reported a high dropout rate as only 17 of 36 patients completed the mailed satisfaction survey [30].

Furthermore, it remains challenging to objectify aesthetic results as examinations or respectively interpretations, even with 3D imaging, are observer-related. Therefore, standardized protocols to report patient’s satisfaction and volume measurements should be included in follow-up examinations.

## 5. Conclusions

Enriched autologous fat grafting offers good long-term results in patients with midface deficiency, in improving facial volume loss and skin quality. Standardized evaluating of facial appearance and of patient’s satisfaction is mandatory to develop and improve successful facial rejuvenation procedures.

## Figures and Tables

**Table 1:** Follow up 6 weeks: Survey of patients undergoing lipofilling for rejuvenation for nasolabial and periorbital folds (*n* = 14).

Patient	Gender/Age	Liposuction Area	PO/Volume (cc) Right/Left	NLF/Volume (Cc) Right/Left
1	w/49	Flanks	1/1	1/1
2	w/42	abdominal	2/2	1/1
3	w/60	Abdominal	2/2	2/2
4	w/47	Abdomen flanks	1.5/1.5	1.5/1.5
5	w/51	abdomen	2,5/2,5	2/2
6	m/44	abdomen	1.5/1.5	1.5/1.5
7	w/60	abdomen	3/3	3/3
8	w/47	abdomen	3/3	2/2
9	w/55	legs	5/5	2/2
10	m/30	Abdomen	2,5/2,5	/
11	w/72	legs	1/1	1.5/1.5
12	w/56	Flanks/axillary region	2,5/2,5	3/3
13	w/69	abdomen	5/5	5/5
14	w/59	abdomen	3/3	/

PO = periorbital; NLF = nasolabial fold.

**Table 2:** Follow up 6 weeks: Wrinkle severity scale (WSS) for different facial regions pre- and postoperative and global aesthetic improvement scale (GAIS).

Patient	WSS NLF Pre OP	WSS NLF Post	WSS PO Pre OP	WSS PO Post OP	GAIS (1.5 Months)
1	1.5	0.5	1.5	1	1
2	2	1	1.5	1	1
3	2	1	1.5	0.5	1
4	2	1	1.5	0.5	1
5	1.5	1	1.5	0.5	1
6	2	1	1.5	0.5	2
7	2	1	1.5	0.5	1
8	2	1	1.5	0.5	1
9	2	1	1.5	0.5	1
10	/	/	1.5	1	0
11	2	1	1.5	1	2
12	1.5	1	1.5	1	1
13	1.5	0.5	1.5	1	1
14	/	/	2	1.5	1

The postoperative WSS for NLF after 12 months was 1.20 ± 0.26 (*n* = 10. range 1 to 1.5). For PO the initial WSS was measured with 1.50 ± 0.00 (*n* = 14;) and after 6 weeks with 0.79 ± 0.32 (*n* = 14; range 0.5–1.5). After 12 months and loss of 4 patients, the score was measured with 1.05 ± 0.15 (*n* = 10; range 1–1.5).

**Table 3:** Follow-up 12 months: Survey of patients undergoing lipofilling for rejuvenation for nasolabial and periorbital folds.

Patient	WSS NLF Pre OP	WSS NLF Post OP	WSS PO Pre OP	WSS PO Post OP	GAIS (>6 Months)
1	1.5	1	1.5	1	1
2	2	1.5	1.5	1.5	1
3	2	1	1.5	1	1
4	2	1	1.5	1	1
5	1.5	1	1.5	1	1
6	2	1.5	1.5	1	2
7	2	1.5	1.5	1	1
8	2	1.5	1.5	1	1
9	2	1.5	1.5	1	1
					
10	/	/	1.5	1	0

WSS = wrinkle severity scale; NLF = nasolabial fold; PO = periorbital region; GAIS = global aesthetic improvement scale.
